# The Relationship Between Fear of Falling and Functional Mobility Among Older Adults

**DOI:** 10.1093/geroni/igaf122.2874

**Published:** 2025-12-31

**Authors:** Leah Houston, Jennifer Piazza, Koren Fisher

**Affiliations:** California State University, Fullerton, Aurora, Colorado, United States; California State University, Fullerton, Fullerton, California, United States; California State University, Fullerton, Fullerton, California, United States

## Abstract

Fear of falling (FOF) affects 58% of older adults, irrespective of fall history. Current research indicates that FOF may have negative health effects, including an increased likelihood of falls, social isolation, and decreased physical activity and performance, including poorer balance. Similarly, functional mobility, particularly gait speed, is considered a significant predictor of disability, mortality, and fall risk in older adults. Despite the negative biopsychosocial effects, FOF is largely excluded from comprehensive geriatric assessment tools and fall prevention interventions for older adults. Thus, the purpose of this study is to investigate the relationship between FOF and functional mobility in community-dwelling older adults. This cross-sectional study conducted at the Center of Successful Aging at California State University, Fullerton examined functional mobility and FOF for 33 older adults (74.68 ± 5.380 years, 70% Female, 30% Male) using the 8 Foot Up-and-Go and the Short Falls Efficacy Scale-International (Short FES-I). Linear regression analysis indicated that a greater fear of falling was predictive of slower gait speeds, even after adjusting for age, gender, total number of comorbidities, and fall history (Beta=0.423, p=.002). Additionally, this model explained 27% of the variance for gait speed, indicating that fear of falling plays a notable role in functional mobility in older adults. These findings suggest that fear of falling may be a significant factor contributing to slower gait speeds in older adults. Thus, to improve the functional mobility of older adults, it is crucial to address FOF in holistic fall prevention and physical activity interventions in this population.

